# Regional Differences in Susceptibiity of Bronchial Epithelium to Mesenchymal Transition and Inhibition by the Macrolide Antibiotic Azithromycin

**DOI:** 10.1371/journal.pone.0052309

**Published:** 2012-12-21

**Authors:** Balarka Banerjee, Michael Musk, Erika N. Sutanto, Stephanie T. Yerkovich, Peter Hopkins, Darryl A. Knight, Suzanna Lindsey-Temple, Stephen M. Stick, Anthony Kicic, Daniel C. Chambers

**Affiliations:** 1 School of Paediatrics and Child Health, the University of Western Australia, Nedlands, Western Australia, Australia; 2 School of Medicine and Pharmacology, the University of Western Australia, Nedlands, Western Australia, Australia; 3 Western Australia Lung Transplant Program, Royal Perth Hospital, Perth, Western Australia, Australia; 4 Department of Respiratory Medicine, Princess Margaret Hospital for Children, Perth, Western Australia, Australia; 5 Telethon Institute for Child Health Research, Centre for Child Health Research, the University of Western Australia, Subiaco, Western Australia, Australia; 6 University of British Columbia, James Hogg Research Centre for Cardiovascular and Pulmonary Research, Vancouver, British Columbia, Canada; 7 Department of Anesthesiology, Pharmacology and Therapeutics, University of British Columbia, Vancouver, British Columbia, Canada; 8 Centre for Asthma and Allergy Research Institute (CAARR) The Lung Institute of Western Australia, Sir Charles Gairdner Hospital, Perth, Western Australia, Australia; 9 School of Medicine, The University of Queensland, Herston, Queensland, Australia; 10 Queensland Lung Transplant Service, The Prince Charles Hospital, Brisbane, Queensland, Australia; University of Kansas Medical Center, United States of America

## Abstract

**Objective:**

Dysregulated repair following epithelial injury is a key forerunner of disease in many organs, and the acquisition of a mesenchymal phenotype by the injured epithelial cells (epithelial to mesenchymal transition, EMT) may serve as a source of fibrosis. The macrolide antibiotic azithromycin and the DNA synthesis inhibitor mycophenolate are in clinical use but their mechanism of action remains unknown in post-transplant bronchiolitis obliterans syndrome (BOS). Here we determined if regional variation in the EMT response to TGFβ1 underlies the bronchiolocentric fibrosis leading to BOS and whether EMT could be inhibited by azithromycin or mycophenolate.

**Methods/Results:**

We found that small and large airway epithelial cells from stable lung transplant patients underwent EMT when stimulated with TGFβ1, however mesenchymal protein expression was higher and loss of epithelial protein expression more complete in small airway epithelial cells. This regional difference was not mediated by changes in expression of the TGFβRII or Smad3 activation. Azithromycin potentially inhibited EMT in both small and large airway epithelial cells by inhibiting Smad3 expression, but not activation.

**Conclusion:**

Collectively, these observations provide a biologic basis for a previously unexplained but widely observed clinical phenomena, and a platform for the development of new approaches to fibrotic diseases.

## Introduction

Lung transplantation is the only viable treatment option for many patients with end stage lung disease, however long term outcomes are compromised by the development of bronchiolitis obliterans syndrome (BOS). Over half the recipients will have developed this complication within 5 years of transplant and it is the major cause of late morbidity and mortality [1]. BOS is characterized physiologically by airflow obstruction [2] and histopathologically by obliteration of the lumen of small airways with fibrotic plugs (obliterative bronchiolitis). The pathologic events which result in small airway fibrosis are unknown but are only partially alloimmune in nature [3], [4]. Incomplete and/or dysregulated epithelial repair following injury is central to its pathogenesis [5]. How dysregulated epithelial repair leads to fibrosis and why the bronchiolar region of the allograft is targeted for fibrotic obliteration when the inciting events are generally non-selective are key unanswered questions.

The source of the increased numbers of activated fibroblasts which cause fibrotic obliteration is also unknown, although emerging evidence implicates epithelial cells in the development of fibrosis [6], [7] through the process of epithelial to mesenchymal transition (EMT) [8]. Apart from BOS, EMT has been postulated to contribute to idiopathic pulmonary fibrosis [9], asthma [10] and fibrosis/remodeling of epithelial surfaces in the cornea [11], liver [12] and kidney [8]. However, research into the role of EMT in BOS has previously only involved large airway epithelial cells [6], [13]. Furthermore, a review of current literature does not reveal any studies that have directly assessed the capacity of primary human small airway epithelial cells to undergo EMT.

Once established, BOS treatment is difficult, although the macrolide antibiotic azithromycin has some therapeutic benefit [14], and is an effective prophylactic agent [15]. The mechanisms behind this clinical efficacy are unclear, but have been postulated to be related to dampening of neutrophilic inflammation [16], although more recently, azithromycin was shown to increase trans-epithelial electrical resistance [17] and prevent disintegration of the tight junction protein zona occludens following exposure of epithelium to *Pseudomonas aeruginosa* [18]. However, its exact mechanism of action remains to be fully elucidated.

Given these observations, we hypothesized that (1) both the large and small airway epithelium from lung transplant patients would undergo EMT with TGFβ1 stimulation but (2) that the response to TGFβ1 would be spatially heterogeneous, favoring the small airways. We further hypothesized that azithromycin may exert its therapeutic effects in part by inhibiting EMT. In testing these hypotheses we generated primary cultures of large and small airway epithelial cells from the same lung transplant patient using established methodologies [19], and compared their response to TGFβ1 stimulation. We show that although EMT can be induced in both small and large airway epithelial cells, the EMT response was greater in the small airway epithelium. Furthermore, we provide evidence that azithromycin when delivered in biologically relevant doses [20], inhibits TGFβ1-induced large and small airway EMT by inhibiting Smad3 production. The regional difference in response to TGFβ1 stimulation and the inhibitory effects of azithromycin help resolve some currently unexplained clinical phenomena, and provide an attractive target for further research.

## Materials and Methods

### Reagents and Materials

Fetal Calf Serum (FCS), RPMI-1640 media, penicillin G, streptomycin sulphate, amphotericin B and L-glutamine were purchased from Invitrogen. Insulin, bovine serum albumin (BSA), hydrocortisone, recombinant human epidermal growth factor (EGF), epinephrine hydrochloride, triiodothyronine, retinoic acid, trypsin, gelatin and gentamycin were obtained from Sigma. Bronchial Epithelium Basal Medium (BEBM) was purchased from LONZA. Ultroser G was supplied from Ciphergen. Collagens S (type I) as well as fibronectin were purchased from Roche. TGFβ1 was purchased from R&D Biosystems. Everolimus was provided by Novartis Pharmaceuticals. Mycophenolate, the active compound in mycophenolate mofetil was provided by Roche Pharmaceuticals. Azithromycin in lyophilized powder form was acquired from Pfizer. All tissue culture plastic ware was obtained from Sarstedt and BD Scientific.

### Patients, Cell Collection and Culture

A total of 24 bronchoscopies were performed in 23 patients (9 male; aged 25 to 60 years (median 50 years)). Patient demographics are summarized in Table 1. All patients were in a stable clinical state, without BOS, infection or rejection and were receiving standard immunosuppression consisting of a calcineurin inhibitor, mycophenolic acid and prednisolone. Primary human epithelial cells were collected from the large and small airways, as previously described [19]. Written informed consent was obtained from each patient prior to their participation in the study in accordance with ethics approvals obtained from the Royal Perth Hospital Human Research and Ethics Committee (Registration: EC2006/021) and the Prince Charles Hospital Medical Research Committee (Registration: EC2843). Briefly, airways were brushed using a cytology brush (Olympus® BC-25105) through the working channel of a bronchoscope (Olympus® Evis EXERA II), during routine surveillance bronchoscopy. The small airways were brushed by extending the brush tip to 2–3 cm from the pleural surface under radiological guidance. Lineage confirmation was performed using expression of Clara Cell Specific Protein (CCSP) and surfactant protein B [19]. Cells were collected in a sterile tube containing RPMI-1640. At least 3–4 passes were made at each region to ensure an adequate cell yield. After all brushings were completed 20% (v/v) FCS was added prior to immediate processing. Monolayer cultures of small airway epithelial cells (SAEC) and large airway epithelial cells (LAEC) were then established as previously described with the only modification that cells were cultured directly in 12 well plates and cultures were not expanded.

**Table 1:** Demographics of patient cohort.

Total number		23
Males (%)		9 (38%)
Median age in years (range)		50 (25–60)
Median time sampled post transplant in months (range)		9 (3–24)
Reason for Transplant		
	*Cystic fibrosis*	6
	*Lymphangioleiomyomatosis*	1
	*Congenital defects*	2
	*Usual interstitial pneumonia*	5
	*COPD*	5
	*α1-antitrypsin deficiency emphysema*	3
	*Sarcoidosis*	1
Type of Transplant		
	*Bilateral*	15
	*Heart-Lung*	3
	*Single*	4
	*Heart-Lung-Liver*	1

### Induction of EMT *in vitro*

Cell cultures were maintained until approximately 60–70% confluent upon which they were incubated in basal medium for 24 h. TGFβ1 (50 ng/ml) was then added and the cells incubated for 96 h. TGFβ1 was then added to the cells at a final dose of 50 ng/ml in starvation basal media and incubated for 96 h. This concentration of TGFβ1 was utilized after trials of three separate doses (1 ng/ml, 10 ng/ml and 50 ng/ml) found 50 ng/ml to induce complete EMT (Fig. S1). Cell lysate and cell culture supernatant were collected at 0 h, 48 h and 96 h for Western blot analysis and zymography respectively.

### Prevention of EMT

Cell cultures were grown until 60–70% confluent. At this point, cells were placed in starvation media for 24 h and then stimulated with TGFβ1 (50 ng/ml) alone or in combination with azithromycin (1–50 µg/ml); mycophenolate (0.05–5 µg/ml) or everolimus (0.01–1 nM) for 96 h. Mycophenolate was dissolved by gentle sonication in 0.86 ml Benzyl Alcohol, 0.37 ml Tween−80, 0.9 g NaCl, 0.5 g carboxymethylcellulose and milliQ water in 100 mL at pH 3.5 as per manufacturer’s recommendation (Roche Pharmaceuticals). Once dissolved, the solution was further diluted with starvation media so that the final concentration of the diluent in culture was not higher than 0.01% (v/v). Everolimus was dissolved in 100% ethanol and diluted in basal media so that the final concentration of ethanol in culture did not exceed 0.01% (v/v). Azithromycin was dissolved in milliQ water. Cell lysate and culture supernatants were collected as described above.

### Western Blot Analysis

Protein lysates were analyzed for expression of EMT markers (zona occludens 1 (ZO-1); EDA-Fibronectin (EDA-Fn); Vimentin (Vim); and Cytokeratin 19 (Ck-19). To test the role of Smad3 in EMT induction and prevention, protein expression of Smad3 and pSmad3 were also measured. Briefly, protein samples (20 µg) were electrophoresed on a 10% 1.0 mm Bis-Tris Sodium Dodecyl Sulphate (SDS) Polyacrylamide gels (Invitrogen) using a NuPage Western Blot apparatus (Invitrogen) at 200V for 45 min. Proteins were transferred onto PVDF membrane (Millipore) using dry transfer method on iBlot (Invitrogen) (200V for 13 min) or wet transfer (230 mA, at 4°C for 2 h). Membranes were then stained for ZO-1 (Invitrogen), EDA-Fn, Vim (AbCam) and Ck-19 (Sigma) as well as β-actin for normalization. Odyssey Licor 800 and 680 secondary antibodies were used for detection. LICOR® Odyssey v3.0 software was used to quantify the bands. The integrated intensity (I.I.) of each band was then normalized to the I.I. of the β-actin band in the corresponding lane.

### Gelatin Zymography

Samples were electrophoresed on SDS-PAGE gels containing gelatin as a substrate for MMP 2 and 9. Briefly, 10% (v/v) Bis-Tris gels with a final concentration of 10% (m/v) gelatin were prepared in the laboratory. Supernatant samples were warmed at 37°C for 5 min before being loaded onto the gel with non-reducing loading buffer, ensuring equal amounts of protein in every lane (25 µg). The gels were electrophoresed for 90 min at 210V at 4°C and then renatured by washing in 0.1% (v/v) Triton-X solution 3 times for 20 min each wash, with gentle agitation. After renaturation, gels incubated in development buffer for 30 min at room temperature (RT), followed by incubation in fresh development buffer at 37°C overnight. The following day, gels were stained in Coomasie blue solution and then destained until clear bands were observable. The gels were visualized using the LICOR Odyssey at 700 nm and bands were quantified using the LICOR Odyssey v3.0 software. The activity of MMPs was measured by densitometric analysis of bands of enzymatic digestion as previously described [5].

### Quantitative RT-PCR of TGFβRII

Gene expression of TGFβRII was assessed in *ex vivo* samples of brushings collected from small and large airways of patients via two-step reverse transcriptase polymerase chain reaction (RT-PCR) according to established methods [21]. Briefly, total cellular RNA was extracted from epithelial cells with RNeasy mini columns (QIAGEN), a DNA digest performed with RNase-Free DNase (QIAGEN) to remove unwanted DNA. Reverse transcription was performed to convert 200 ng of RNA into cDNA: 200 ng of RNA was added to a master mix containing 10×RT Buffer (2 µl), 5 mM MgCl_2_ (4.4 µl), 2 mM deoxyribonucleotide triphosphates (dNTPs) (1.0 µl), Random Hexamers (1.0 µl), RNase Inhibitor (0.4 µl), and MultiScribe (0.5 µl) and then made to a final volume of 20 µl with RNase free water. Samples were then placed in a PTC-100 Thermal Cycler (MJ Research) and run on a standard reverse transcription program consisting of 30 cycles: 92°C for 30 seconds followed by 3 minutes at 60°C. Primers from previously published sequences for the gene of interest (TGFβRII) [22] and the house keeping gene (PPIA) [23] were obtained from GeneWorks. The RT-qPCR reactions contained cDNA (10 ng), forward and reverse primers (0.3 µM) for TGFβ1 RII (Forward: 3′-CATGACCCCAAGCTCCCCTAC-5; Reverse: 5′-CATGAAGAAAGTCTCACCAGG-3′) or PPIA for normalization (Forward: 3′-TGAGCACTGGAGAGAAAGGA-5′; Reverse: 5′-CCATTATGGCGTGTAAAGTCA-3′), SYBR®GREEN PCR Master Mix (10 µl) and RNase free water to make a final volume of 20 µl. RT-qPCR was performed on an AB-7300 analyzer (Applied Biosystems).

### Immunocytochemical Analysis of TGFβRII Expression

Large and small airway epithelial cells cytospun on glass microscope slides, were stained for TGFβRII expression. Briefly, epithelial cells cytospun onto slides were fixed using 10% neutral buffered formalin for 20 min RT. Antigen retrieval was performed on the fixed slides by immersing them in 0.01 M sodium citrate buffer (pH 6.0) and heating in a microwave oven 15 min. Autofluoroscence of the cells was blocked by incubating slides in 0.5% (w/v) Sudan B Black solution in 70% (v/v) ethanol for 20 min. The slides were washed with PBS containing 1% (v/v) saponin. Cells were then blocked for 1 hour at RT in 5% (w/v) BSA, 10% (v/v) FCS, 0.1% (v/v) Triton X-10 in PBS/saponin solution. Slides were stained for primary antibody for TGFβRII (BD Scientific) (1∶200) and incubated for 18 h at 4°C. Slides were then washed in PBS/Saponin and stained for secondary antibody AlexaFluor 488 (Invitrogen) (1∶1000) for 18 h at 4°C. Cells were washed again in PBS/Saponin and the nuclear stain DAPI (Sigma) (1∶50000) was applied. Fluorescent antibody complexes were then visualized using a fluorescent microscope (Nikon).

### Flow Cytometric Analysis of TGFβRII Expression

TGFβRII expression (as assessed by mean fluorescence intensity, MFI) was measured on small and large airway epithelial cells using flow cytometry. Briefly, cells were re-suspended in RPMI supplemented with 10% v/v fetal calf serum prior to three passes through a 25G needle to create a single cell suspension. Single cells were added to FACS tubes and washed with 1% v/v FCS in PBS. Cells were then incubated for 20 min with TGFβRII-PE (US Biologics), washed and fixed in 1% v/v paraformaldehyde. The cells were then permeabilized with commercially available permeabilization buffer (BD Biosciences) and stained with cytokeratin-FITC (Miltenyi Biotech) to confirm epithelial origin. Samples were collected on a FACSCanto (BD Biosciences) and analysed using FlowJo software (Tree Star Inc.).

### Statistical Analysis

All data were assumed to be non-parametric, with analysis performed using the Wilcoxon signed rank test. Data presented are median ± interquartile range (IQR). Significance level taken at α = 0.05.

## Results

### TGFβ1-induced EMT is Enhanced in Allograft Small Airway Epithelium

**Figure 1:**
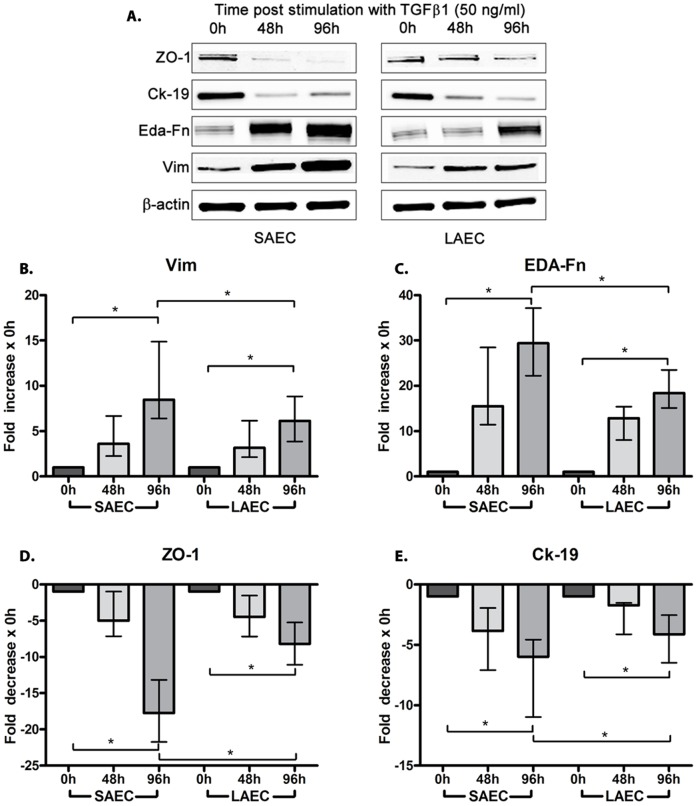
Changes in expression of epithelial and mesenchymal proteins in primary human small airway epithelial cells (SAEC) and large airway epithelial cells (LAEC) stimulated with TGFβ1 *in vitro.* Both SAEC and LAEC cell cultures were stimulated with TGFβ1 (50 ng/ml) for 96 h and cellular protein was collected and analyzed by Western blots. (A) Initial immunoblots suggested a marked increase in mesenchymal markers and corresponding decrease in epithelial markers after 96 h of stimulation. Quantification of immunoblots showed that there was significantly (*p<0.05) increased expression of the mesenchymal markers (B) Vimentin (Vim) and (C) EDA-Fibronectin (EDA-Fn) as well as significantly decreased expression of epithelial markers (D) Zona Occludens -1 (ZO-1) and (E) Cytokeratin-19 (Ck-19) (all normalized to β-actin). SAEC was also found to be more susceptible to EMT as the increase in mesenchymal markers and decrease in epithelial markers was significantly greater for SAEC compared to LAEC after 96 hours of TGFβ1 stimulation (p<0.05, n = 23). Data presented as median ± IQR.

**Figure 2:**
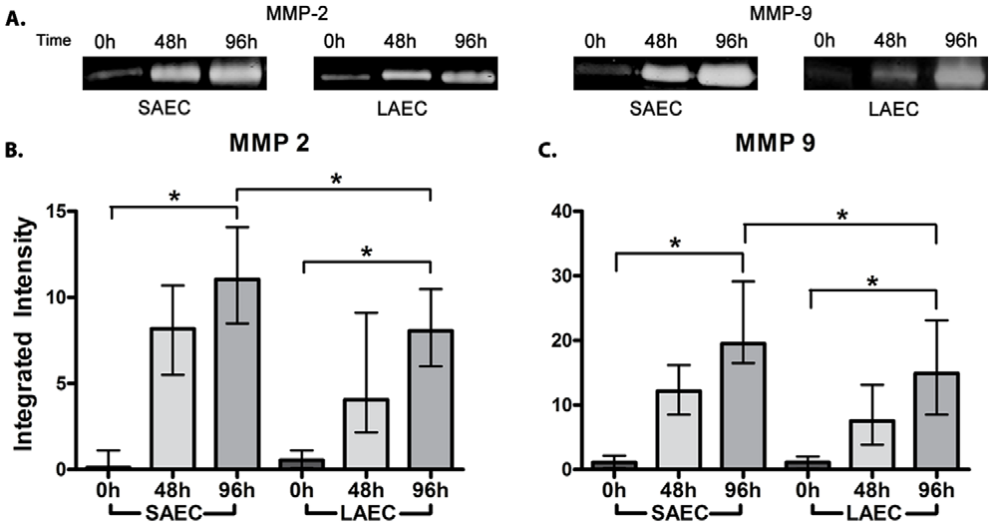
Changes in activity of MMP-2 and -9 in primary human small airway epithelial cells (SAEC) and large airway epithelial cells (LAEC) stimulated with TGFβ1 *in vitro*. Both SAEC and LAEC cell cultures were stimulated with TGFβ1 (50 ng/ml) for 96 h and activity of secreted matrix metalloproteinase (MMP) −2 and −9 in the culture supernatant was measured by gelatin zymography. (A) Initial zymograms suggested a marked increase in the activity of MMP-2 and MMP-9 after 96 h of stimulation. Densitometric quantification of zymograms showed a significant increase in the activity of (B) MMP-2 & (C) MMP-9 in the supernatant in both SAEC and LAEC. Furthermore, the increase in MMP-2 and -9 activity was significantly higher in SAEC compared to LAEC at 96 hrs. (p = 0.0027 for MMP-2 and p = 0.0004 for MMP-9, n = 23). Data presented as median ± IQR.

Following TGFβ1 stimulation, epithelial cell morphology changed from polygonal to spindle-shaped over 96 h (Fig. S2). Co-incident with these morphological changes, we observed a significant time-dependent decrease in the expression of the epithelial proteins ZO-1 (17.7 fold for SAEC & 8.2 fold for LAEC) and Ck-19 (5.9 fold for SAEC & 4.1 fold for LAEC) (Fig. 1). In contrast, expression of the mesenchymal proteins Vim (8.5 fold for SAEC & 6.2 fold for LAEC) and EDA-Fn (29.4 fold for SAEC & 18.4 fold for LAEC) (p<0.05 for all) (Fig. 1) were significantly increased. Using, gelatin zymography we show a significant increase in the activity of MMP-2 (11.1 fold for SAEC & 8.1 fold for LAEC) and MMP-9 (19.5 fold for SAEC & 14.9 fold for LAEC) (p<0.0001 for all) in cell culture supernatant at 96 hours post TGFβ1 stimulation (Fig. 2). In all cases, the EMT response was greater in SAEC compared to patient matched LAEC (p<0.01 for ZO-1, Ck-19, EDA-Fn. MMP-2 and MMP-9, and p = 0.01 for Vim, Fig. 1 and 2).

### Enhanced Response of Small Airway Epithelium to TGFβ1 Stimulation is Not Mediated by Differential Expression of the TGFβ Receptor II or Differential Smad3 Activation

**Figure 3:**
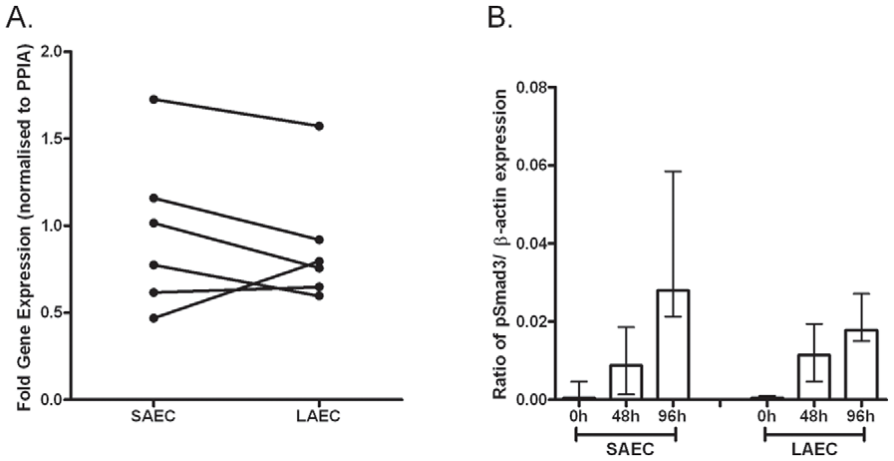
The role TGFβ receptor II (TGFBRII) and of Smad3 activation as potential mechanisms underlying the differential susceptibility of small airway epithelial cells (SAEC) and large airway epithelial cells (LAEC) to TGFβ1 induced EMT. (A) To test whether the increased susceptibility of SAEC to EMT due to an increased expression of TGFBRII, *ex vivo* samples from small and large airway brushings were analyzed for gene expression of TGFBRII qRT-PCR. No significant difference in TGFβRII expression between SAEC and LAEC was observed (p = 0.56) (n = 6). (B) To investigate if increased susceptibility of SAEC to EMT was due to differences in the activation of the Smad-dependent pathway, the rate of Smad3 phosphorylation (normalized to β-actin) post TGFβ1 stimulation was measured by Western blot analysis on cell lysates from stimulated SAEC and LAEC cultures. There was also no significant difference in the rate of Smad3 phosphorylation following TGFβ1 stimulation (p = 0.21, n = 6). Data represented as median ± IQR.

We next sought to understand the mechanism underlying this differential response to TGFβ1 stimulation. First we measured TGFβRII expression in cells *ex-vivo*, and found no significant difference in TGFβRII gene expression between SAEC and LAEC (Fig. 3A). Using immunocytoochemistry and flow cytometry we further found that there was no significant difference in TGFBRII protein expression (Fig. S3). Since TGFβ1 induced EMT is Smad3 dependent [10], we next measured expression of Smad3 and pSmad3 in SAEC and LAEC stimulated with TGFβ1. While TGFβ1 rapidly activated Smad3, the time course of Smad3 phosphorylation was similar in SAEC and LAEC (Fig. 3B).

### Azithromycin Inhibits TGFβ1-induced EMT

**Figure 4:**
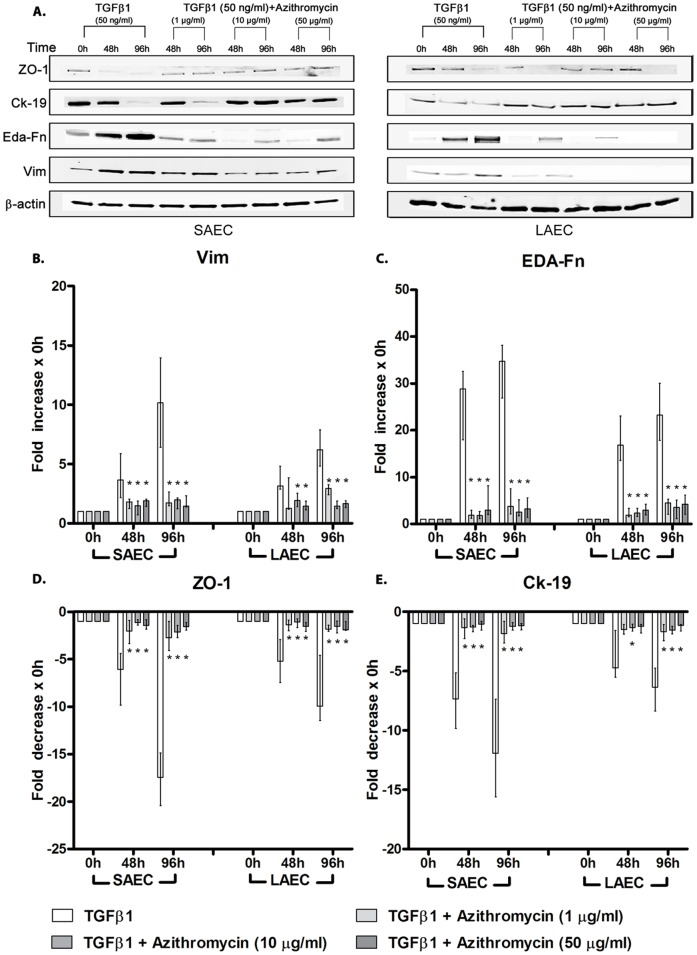
Effect of Azithromycin on TGFβ1-induced EMT of primary human small and large airway epithelial cell cultures – expression of epithelial and mesenchymal markers. Primary small airway epithelial cell (SAEC) and large airway epithelial cell (LAEC) cultures were stimulated with TGFβ1 (50 ng/ml) for 96 h to induce EMT. Azithromycin (1 µg/ml, 10 µg/ml, 50 µg/ml) was simultaneously added to observe its effect of preventing or slowing EMT, by measuring change in expression of epithelial markers (ZO-1, Ck-19) and mesenchymal markers (EDA-Fn, Vim) by Western blots. (A) Initial immunoblots suggested that addition of azithromycin even at a sub-clinical dose (1 µg/ml) significantly suppressed EMT compared to the control. Further quantification of the immunoblots showed a suppression in theincrease in expression of mesenchymal markers (B) vimentin (Vim) and (C) EDA- Fibronectin (EDA-Fn), and the decrease in expression of epithelial markers (D) Zona Occludens -1 (ZO-1) and (E) Cytokeratin-19 (Ck-19) in SAEC and LAEC, observed in the control samples which were stimulated with TGFβ1 alone (n = 6). Data presented as median ± IQR. *p<0.05 compared to control.

**Figure 5:**
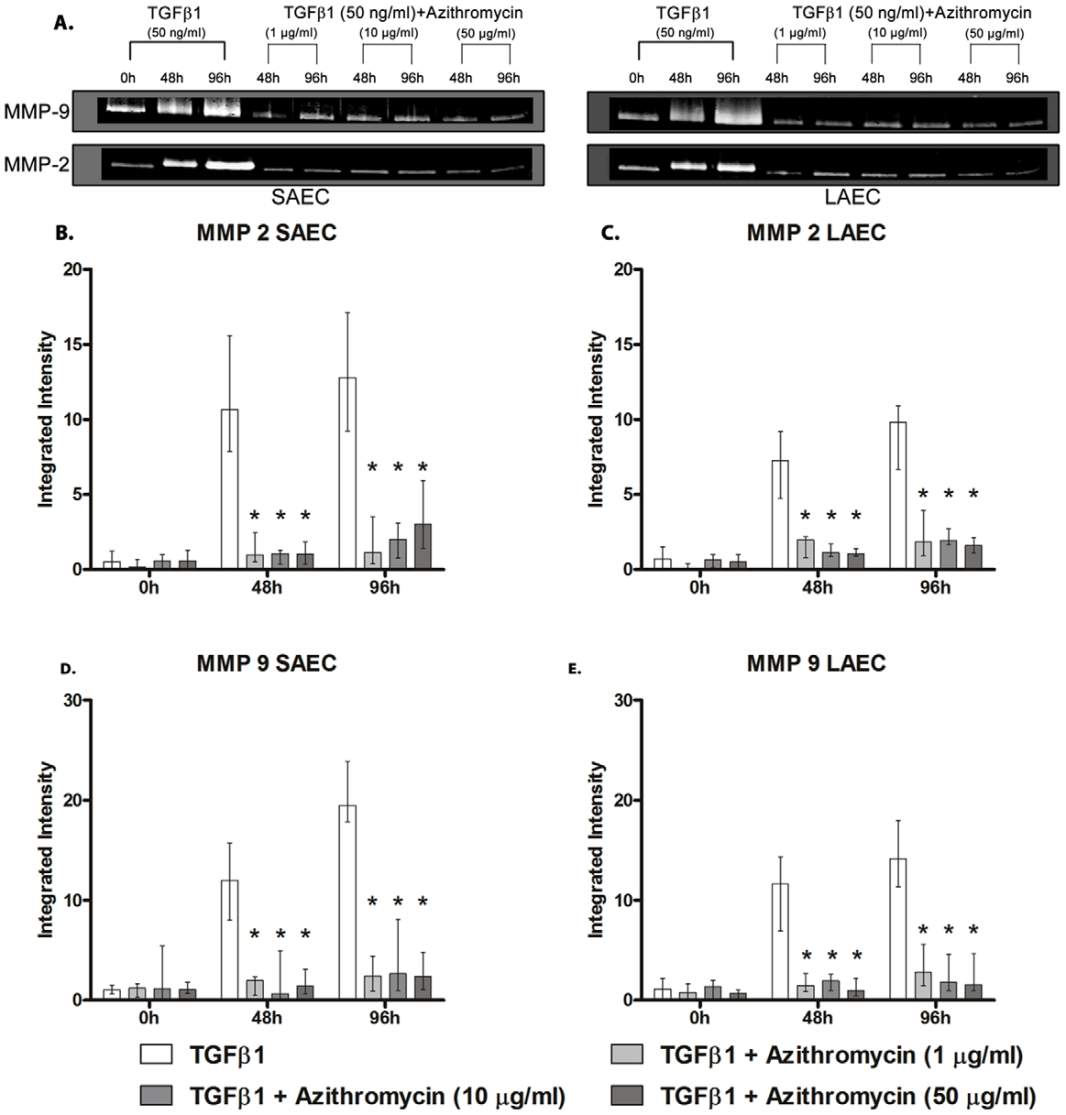
Effect of Azithromycin on TGFβ1-induced EMT of primary human small and large airway epithelial cell cultures - activity of matrix metalloproteinases (MMP) -2 & -9. Primary small airway epithelial cell (SAEC) and large airway epithelial cell (LAEC) cultures were stimulated with TGFβ1 (50 ng/ml) for 96 h to induce EMT. Azithromycin (1 µg/ml, 10 µg/ml, 50 µg/ml) was simultaneously added to observe its effect on preventing or slowing EMT, by measuring associated increase in the gelatinolytic activity of MMP-2 and -9, by gelatin zymography. (A) Initial zymograms suggested that addition of azithromycin even at a sub-clinical dose (1 µg/ml) suppressed EMT compared to the control. Further quantification of thezymograms showed a suppression in the increase in the activity of matrix metalloproteinase (B) (MMP) -2 and (C) MMP -9 in SAEC and LAEC, observed in the control samples which were stimulated with TGFβ1 alone (n = 6). Data presented as median ± IQR. *p<0.05 compared to control.

We next compared pharmaceuticals with potential activity in BOS (azithromycin, mycophenolate and everolimus) for their relative ability to inhibit EMT. We found that azithromycin completely abolished the increase in expression of mesenchymal markers Vim and EDA-Fn even at the lowest concentration studied (1 µg/ml) and simultaneously preserved expression of the epithelial markers Ck-19 and ZO-1 (Fig. 4). Changes in MMP-2 and -9 activity were also abolished (Fig. 5). EMT was also completely abolished at the higher doses studied (10 µg/ml and at 50 µg/ml).

The DNA synthesis inhibitor mycophenolate was a less effective inhibitor of EMT, with a modest reduction in mesenchymal protein expression, at a concentration of 5.0 µg/ml (Fig. S4), and less complete preservation of expression of Ck-19 and ZO-1 (Fig. S5). The effects of mycophenolate on MMP-2 and -9 activity were also consistent with it being a less effective inhibitor of EMT (Fig. S5). In contrast, the mammalian target of rapamycin (mTOR) inhibitor everolimus had no significant effect on TGFβ1-induced EMT at any of the concentrations studied (Fig. S6 and S7).

### Azithromycin Inhibition of TGFβ1-induced EMT is Smad3 Dependent

**Figure 6:**
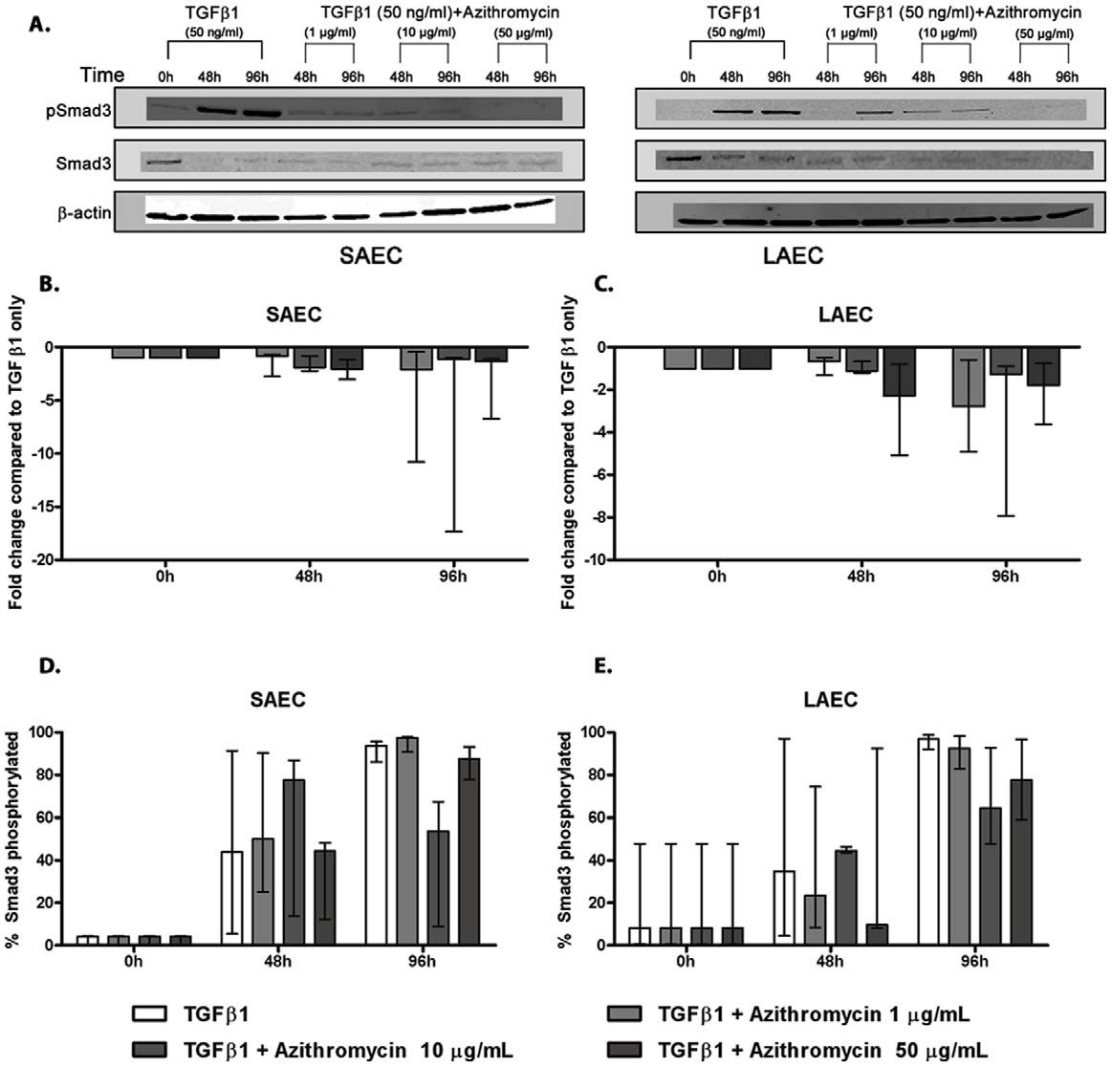
Mechanism of effect of Azithromycin on preventing TGFβ induced EMT – Smad3 production and phosphorylation. Primary small airway epithelial cell (SAEC) and large airway epithelial cell (LAEC) cultures were stimulated with TGFβ1 (50 ng/ml) for 96 h to induce EMT. Azithromycin (1 µg/ml, 10 µg/ml, 50 µg/ml) was simultaneously added to observe its effect of preventing or slowing EMT. (A) Expression of Smad3 and pSmad3 was then measured by Western blot analysis and the immunoblots were further quantified by densitometric analysis. (B) Addition of azithromycin even at a sub-clinical dose (1 µg/ml) suppressed the expression of total Smad3 in SAEC and LAEC, compared to the control cultures stimulated with TGFβ1 alone. (C) Addition of azithromycin (1 µg/ml, 10 µg/ml, 50 µg/ml) did not alter the rate of Smad3 phosphorylation in SAEC and LAEC, compared to the control. (n = 3) Data presented as median.

Having shown such a pronounced inhibitory effect of azithromycin, we attempted to determine the mechanism by measuring Smad3 production and activation in azithromycin treated cells. Azithromycin (1 µg/ml) led to very low total Smad3 levels within 48 hours, while the proportion of Smad3 which was phosphorylated was not different from untreated control cells at any time point (Fig. 6). Comparable effects were seen at higher doses. A similar trend was noted for mycophenolate stimulated samples (Fig. S8), although the inhibition of Smad3 production was greater for azithromycin.

## Discussion

In this study we have demonstrated that both small and large airway epithelial cells from stable lung transplant patients are capable of undergoing EMT when stimulated with TGFβ1. More significantly, the EMT response was greater in SAEC, an effect not mediated by changes in expression of TGFβRII or by changes in Smad3 activation. Comparison of three pharmaceuticals with potential activity in BOS revealed that azithromycin was a potent inhibitor of EMT in both SAEC and LAEC, while mycophenolate was a weaker inhibitor and the mTOR inhibitor, everolimus, had no effect. We show that the effect of azithromycin was mediated through inhibition of Smad3 expression, with no effect on Smad3 activation.

EMT has been implicated in the pathogenesis of multiple fibrotic conditions and provides an attractive framework for understanding the steps leading to organ failure as well as providing the opportunity for the development of novel therapeutic agents. Since BOS is associated with dysregulated epithelial repair [13] and marked intragraft upregulation of TGFβ1 [24], EMT as a source of myofibroblasts eventually causing airway obliteration is a logical hypothesis and is supported by previous work [6], [7]. While recent fate-mapping studies [25], [26] have challenged the role of EMT in lung and liver fibrosis respectively, the animal models of acute fibrosis employed in these studies are poorly reflective of human fibrotic disease which usually occurs in a multi-step, chronic fashion, and contradict multiple human studies demonstrating co-localization of epithelial and mesenchymal markers in transitioning epithelium, as recently reviewed [27]. Our study, as the first to study human lung transplant small airway epithelium, adds further weight to the argument that EMT plays a role in human lung allograft fibrosis, since our *in vitro* observations help explain the preferential involvement of the small airway region of the allograft by fibrosis and also the therapeutic effect of azithromycin.

While our data are the first to demonstrate regional differences in the capacity for EMT to occur in the human bronchial tree, there is strong support from existing *in vitro* and animal data for the idea that there is a progressive increase in susceptibility to EMT with distance from the central airways [28], [29]. EMT is easily induced by *in vitro* TGFβ1 stimulation of the human alveolar cell line A549 [29], but not by stimulation of bronchial cell lines such as 16HBE4o^−^ [28], while bleomycin induces EMT in murine alveolar and bronchiolar epithelial cells, but not corresponding bronchial cells [30]. However, this data is based solely on immortalized cell lines and murine models and corresponding experiments on primary human tissue have not been previously performed. We suspected that the differential susceptibility may be attributed to a higher concentration of TGFβRII on the small airway epithelia of transplant patients since the amount of this receptor varies depending on the cell type and is also influenced by disease conditions [31], [32]. Activation of TGFßRII and TGFβRI by TGFβ1 is the first essential step in the induction of canonical pathways, which may be Smad-dependent or -independent. However, we found no significant difference in TGFβRII expression. We also found no difference in the magnitude of activation of Smad3. Our data suggest that the increased capacity of small airway epithelium to undergo EMT is either related to alterations in the TGFβ1 signalling cascade downstream of Smad3 or occurs via a non-canonical TGFβ1 signalling pathway.

There is now a substantial body of clinical data demonstrating the efficacy of azithromycin both in the treatment and prevention of BOS [33]–[36], but only limited data for mycophenolate [37], [38] and everolimus [39]. While it is clear that the mechanism of action of azithromycin is not related to antibacterial activity, the precise mode of action has remained elusive [16], [40]. For the first time, we have shown that the therapeutic activity of azithromycin in airway diseases may be related to inhibition of EMT and the maintenance/preservation of epithelial phenotype. This effect was seen even at a concentration (1 µg/ml) well below that achieved in clinical practice. After chronic use, the concentration of azithromycin in cystic fibrosis sputum ranges from 12 to 53 µg/ml and is still detectable at concentrations in the range of 4 to 27 µg/ml 10 days after the last dose [20].

While we have shown that azithromycin inhibits Smad3 expression it remains to be seen whether this regulation occurs at the transcriptional or post-transcriptional level. To our knowledge the effect of azithromycin on the production or activation of Smad3 or other components of the TGFβ1 pathway has not previously been studied, however the erythromycin derivative EM703, may be able to decrease Smad mRNA levels [41]. Furthermore, while a similar function has not previously been described in mammals, the regulation of protein synthesis at a ribosomal level accounts for some of the anti-microbial properties of azithromycin [42], [43].

Our data regarding the role of azithromycin in the maintenance of epithelial phenotype are in line with those of Asgrimsson *et al.* who showed that azithromycin treatment increased transepithelial electrical resistance [17], probably through effects on tight-junctions, and those of Halldorsson *et al*. who showed that azithromycin attenuated the loss of tight junctions and cellular polarity induced by *P. aeruginosa* via an undetermined pathway [18]. Furthermore, *P. aeruginosa* has been implicated in BOS pathogenesis [4] through its ability to accentuate TGFβ1-induced EMT [44]. While the effect of macrolide antibiotics on Smad signalling has received little attention, erythromycin and its synthetic derivative EM703 are able to inhibit fibroblast activation via a Smad-dependent pathway [41]. Taken together, this body of work demonstrates that azithromycin may be effective in airway disease by inhibiting Smad-dependent EMT and so favoring the restoration of the epithelial phenotype after airway injury.

In addition to macrolide antibiotics including azithromycin, DNA synthesis inhibitors such as mycophenolate have also been trialed for their efficacy in preventing chronic rejection in renal transplant patients. Although an initial phase III randomized trial showed no significant improvement in graft survival at 12 months [45], a study by Ojo and colleagues [46] which assessed the US transplant registry data over a 10 year period revealed a 27% lower risk of chronic renal allograft failure independent of acute rejection [46]. The effects of mycophenolate in preventing acute rejection through potent suppression of T- and B-cell suppression in multiple organs has been well established [47], [48]. However, with regard to chronic lung allograft dysfunction, only limited uncontrolled clinical trials have been performed, with mycophenolate being shown to have an effect on stabilizing and slowing FEV1 decline associated with BOS progression [38], [49]. Furthermore, an *in vitro* study has shown that mycophenolate can inhibit the pulmonary fibroblast proliferation [50].

In the current study, mycophenolate significantly inhibited EMT at clinically achievable concentrations [50], [51]. Our findings are in line with previous studies which have demonstrated inhibition of fibroblast activation by mycophenolate through as yet undetermined pathways independent of its immunosuppressive effects [52], [53]. Since fibroblast activation in response to TGFβ1 is Smad-dependent [54] and since mycophenolate can lower renal Smad3 expression at the gene level [55], our data suggest that mycophenolate may act to prevent EMT through inhibition of Smad3 expression. Notwithstanding the efficacy of mycophenolate, the magnitude of effect was lower than that seen with azithromycin even when the latter was delivered at a subtherapeutic concentration (1 µg/ml).

Everolimus has been studied in the context of lung transplantation and has been of interest not only for its immunosuppressive, but also its antifibrotic and antitumor effects [56], [57] Although the antitumor effects of everolimus,are mediated in part through effects on the PI3/Akt TGFβ1 signalling pathway [58], Smad phosphorylation [59] and Snail expression [60], we found no effect of everolimus on TGFβ1-induced EMT. While this lack of effect was somewhat surprising, the antifibrotic effect of everolimus has been shown to be mediated by post-transcriptional control of collagen gene expression rather than by effects on Smad signalling [57], The lack of effect of everolimus in our *in vitro* model highlights the fundamental difference between EMT leading to carcinogenesis and EMT leading to fibrosis.

We acknowledge a number of limitations to this study. The use of primary human airway material and the concurrent establishment of both large and small airway cultures limited our ability to assess a larger array of EMT markers or to perform migration assays. Further studies should also focus on the effects of azithromycin on gene expression of key TGF signaling pathway proteins. Due to ethical constraints around the small airway brushing procedure we were also unable to include a non-transplant healthy control group. Our now established evidence base for the safety of this procedure will facilitate such recruitment in future studies. While our data have been generated using an *in vitro* model, they likely more closely reflect the *in vivo* situation as we have used primary cells and have minimized *ex vivo* manipulation by proceeding to TGFβ1 stimulation without expanding the cells in culture and minimizing the time spent in culture (7–10 days) before stimulation. There is a number of concerns regarding some of the commonly used *in vivo* model such as heterotopic tracheal transplantation, regarding their, reproducibility, relevance and validity to the clinical condition [61], [62]. However, by using primary epithelial cells from living allografts, we believe our model presents critical advantages in relevance compared to mouse or rat models. We have deliberately maintained our cells in a submerged, undifferentiated (basal) state, as the establishment of differentiated cultures at air-liquid interface (ALI) would necessitate downstream experimentation on quite different large and small airway cellular phenotypes (differentiated small airway epithelium is, for instance, non-ciliated), and as small airway epithelial ALI cultures have never before, to our knowledge, been established. Additionally, previous investigators have shown that it is the basal cell layer which is most susceptible to EMT [10]. Since one of the main aims of our study was to investigate the prevention of EMT by candidate pharmacologics, we elected to use a relatively high dose of TGFβ1 (50 ng/ml) to ensure that the cells undergo complete EMT within 96 hours. Our optimization experiments demonstrated the dose-response for TGFβ1-induced EMT over a dose range of 1–50 ng/ml and confirmed the absence of detrimental effects on the cell culture even at the highest dose (Fig S1).

Fibrosis is a significant clinical problem as it is a common cause of end-stage disease in multiple organs and, in contrast to inflammatory pathologies, is largely untreatable. BOS is a typical example of a fibrotic process which poses an almost insurmountable problem for patients and transplant physicians as it is usually fatal without re-transplantation. In this regard, our findings are of particular clinical significance as they provide a mechanistic framework for understanding BOS pathogenesis and, at the same time, provide insight into the observed clinical phenotype and the mechanism of action of the only drug with proven clinical efficacy. One implication of our findings is that azithromycin may be an effective agent for fibrosis in other diseases and organs as has been recently shown [63].

In conclusion, utilizing a novel primary airway cell culture model, we have demonstrated that allograft small airway epithelium has a greater capacity to undergo EMT, perhaps explaining the BOS phenotype. Furthermore, and of significance, we have shown that the macrolide azithromycin and the DNA synthesis inhibitor mycophenolate inhibit human airway EMT at biologically relevant concentrations.

## Supporting Information

Figure S1 The ability of TGFβ1 to induce EMT in the primary human allograft SAEC and LAEC was compared at 3 different concentrations of TGFβ1–1 ng/ml, 10 ng/ml and 50 ng/ml (n = 4). The change in the expression of mesenchymal markers (A) Vimentin (Vim), (B) EDA- Fibronectin (EDA-Fn) and epithelial marker (C) Zona Occludens -1 (ZO-1), (D) Cytokeratin-19 (Ck-19) was measured by Western blots and change in (E) MMP -2 and (F) MMP -9 activity was measured by gelatin zymography. At 96 h post stimulation there was a decrease in the expression of epithelial markers, and increase in the expression of mesenchymal markers as well as MMP-2 and −9, as typically observed during EMT for all doses studied. A clear dose-response relationship was observed, with the most pronounced change in protein expression observed when cultures were stimulated with 50 ng/ml of TGFβ1. No cell loss was observed for any of the cultures. For future studies 50 ng/ml of TGFβ1 was chosen as the optimal concentration to induce EMT after 96 h of *in vitro* stimulation in SAEC and LAEC. Data presented as median ± IQR. *p<0.05
(TIF)

Figure S2 Morphological changes in small airway epithelial cells (SAEC) and large airway epithelial cells (LAEC) when stimulated with TGFβ1 (50 ng/ml) for 96 h *in vitro.* SAEC and LAEC cell cultures were stimulated with TGFβ1 and were photographed using phase contrast microscopy at 0 h, 48 h and 96 h. Unstimulated (left column) SAEC (top row) and LAEC (bottom row) exhibit typical polygonal, cobblestone morphology. Upon stimulation with TGFβ1, cells were observed to elongate and become spindle-shaped. After 96 h, cells assumed the typical mesenchymal phenotype, consistent with epithelial-mesenchymal transition (EMT). (400x magnification).
(TIF)

Figure S3 To test whether the increased susceptibility of SAEC to EMT was due to an increased expression of TGFβRII, *ex vivo* samples from small and large airway brushings were analysed for protein expression of TGFβRII using immunocytochemistry (A, n = 6, 400x magnification) and flow cytometry (B&C, n = 8). Figure S3B demonstrates the mean fluorescent intensity (MFI) for TGFβRII, and Figure S3C demonstrates the proportion of epithelial cells expressing TGFβRII. For immunocytochemistry epithelial cells were cytospun onto glass slides and stained for TGFβRII expression (green, indicated by arrows) as well as the nuclear stain DAPI (blue). For flow cytometry, cells were stained with, TGFβRII-PE and counterstained with cytokeratin-FITC to confirm epithelial lineage. There was no difference in either the intensity of expression or the proportion of epithelial cells expressing the TGFβRII.
(TIF)

Figure S4 Effect of Mycophenolate on TGFβ1-induced EMT of primary human small and large airway epithelial cell cultures – expression of epithelial and mesenchymal markers. Primary small airway epithelial cell (SAEC) and large airway epithelial cell (LAEC) cultures were stimulated with TGFβ1 (50 ng/ml) for 96 h to induce EMT. Mycophenolate (5 µg/ml) was simultaneously added to observe its effect of preventing or slowing EMT. (A) Initial immunoblots suggested that addition of mycophenolate suppressed EMT in a dose dependant manner. Further quantification of immunoblots showed a marked increase in mesenchymal markers (B) Vimentin, (C) EDA-Fibronectin and corresponding decrease in epithelial markers (D) Zona Occludens -1, (E) Cytokeratin-19 after 96 h of stimulation. Addition of mycophenolate at the highest dose (5 µg/ml) significantly suppressed the increase in expression of mesenchymal markers and the decrease in expression of epithelial markers in SAEC and LAEC, observed in the control samples which were stimulated with TGFβ1 alone (n = 6). Data presented as median ± IQR. *p<0.05 compared to control.
(TIF)

Figure S5 Effect of Mycophenolate on TGFβ1-induced EMT of primary human small and large airway epithelial cell cultures - activity of matrix metalloproteinases (MMP) -2 & -9. Primary small airway epithelial cell (SAEC) and large airway epithelial airway cell (LAEC) cultures were stimulated with TGFβ1 (50 ng/ml) for 96 h to induce EMT. Mycophenolate (5 µg/ml) was simultaneously added to observe its effect on preventing or slowing EMT. (A) Initial zymograms suggested a suppression of EMT in a dose dependant manner. The suppression of EMT associated increase in gelatinolytic activity of (B&C) MMP-2 and (D&E) MMP-9 by mycophenolate was further quantified. Addition of mycophenolate at the highest dose (5 µg/ml) suppressed the increase in the activity of matrix metalloproteinase (MMP) 2 and 9 in SAEC and LAEC, observed in the control samples which were stimulated with TGFβ1 alone (n = 6). Data presented as median ± IQR. *p<0.05 compared to control.
(TIF)

Figure S6 Everolimus was unable to prevent TGFβ1 (50 ng/ml)-induced of small airway epithelial cell (SAEC) and large airway epithelial cell (LAEC) cultures. Primary small (SAEC) and large (LAEC) airway epithelial cell cultures were stimulated with TGFβ1 (50 ng/ml) for 96 h to induce EMT. Everolimus was simultaneously added to observe its effect of preventing or slowing EMT. (A) Initial immunoblots suggested that addition of everolimus at all three doses (0.01 nM, 0.1 nM, 1 nM) did not significantly suppress EMT. Further quantification of immunoblots showed differences in expression of mesenchymal markers (B) Vimentin, (C) EDA-Fibronectin and epithelial markers (D) Zona Occludens -1, (E) Cytokeratin-19 after 96 h of stimulation, compared to control samples which were stimulated with TGFβ1 alone. (n = 6) Data presented as median ± IQR. *p<0.05 compared to control.
(TIF)

Figure S7 Addition of everolimus had no significant effect on the increase in the activity of matrix metalloproteinases (MMP) 2 & 9 associated with EMT. Primary small airway epithelial cell (SAEC) and large airway epithelial cell (LAEC) cultures were stimulated with TGFβ1 (50 ng/ml) for 96 h to induce EMT. Everolimus was simultaneously added to observe its effect on preventing or slowing EMT. (A) Initial zymograms suggested no suppression of EMT. The change in gelatinolytic activity of (B&C) MMP-2 and (D&E) MMP-9 by mycophenolate was further quantified. Addition of everolimus at all three doses (0.01 nM, 0.1 nM, 1 nM) did not significantly change the increase in the activity of matrix metalloproteinase (MMP) 2 and 9 in SAEC and LAEC, compared to control samples which were stimulated with TGFβ1 alone (n = 6). Data presented as median ± IQR. *p<0.05 compared to control.
(TIF)

Figure S8 Mycophenolate partially prevented EMT by suppressing the expression of Smad3, but not Smad3 phosphorylation. Primary small (SAEC) and large (LAEC) airway epithelial cell cultures were stimulated with TGFβ1 (50 ng/ml) for 96 h to induce EMT. Mycophenolate was simultaneously added to observe its effect of preventing or slowing EMT. (A) Protein expression of Smad3 and pSmad3 was measured by Western blots. (B) Addition of mycophenolate (0.05 µg/ml, 0.5 µg/ml, 5 µg/ml) partially suppressed the expression of total Smad3 (Smad3+ pSmad3) in SAEC and LAEC, relative to the control cultures stimulated with TGFβ1 alone. This effect was particularly noticeable at the highest dose (5 µg/ml) (C) However addition of mycophenolate at all doses (1 µg/ml, 10 µg/ml, 50 µg/ml) did not alter the rate of Smad3 phsophorylation compared to TGFβ1 alone (n = 3) Data presented as median ± IQR.
(TIF)
