# Supplementary figures and images for: Regional Differences in Susceptibiity of Bronchial Epithelium to Mesenchymal Transition and Inhibition by the Macrolide Antibiotic Azithromycin
Source: PLoS One. 2012 Dec 21;7(12):e52309. doi: 10.1371/journal.pone.0052309 (PMC3528745; doi:10.1371/journal.pone.0052309)

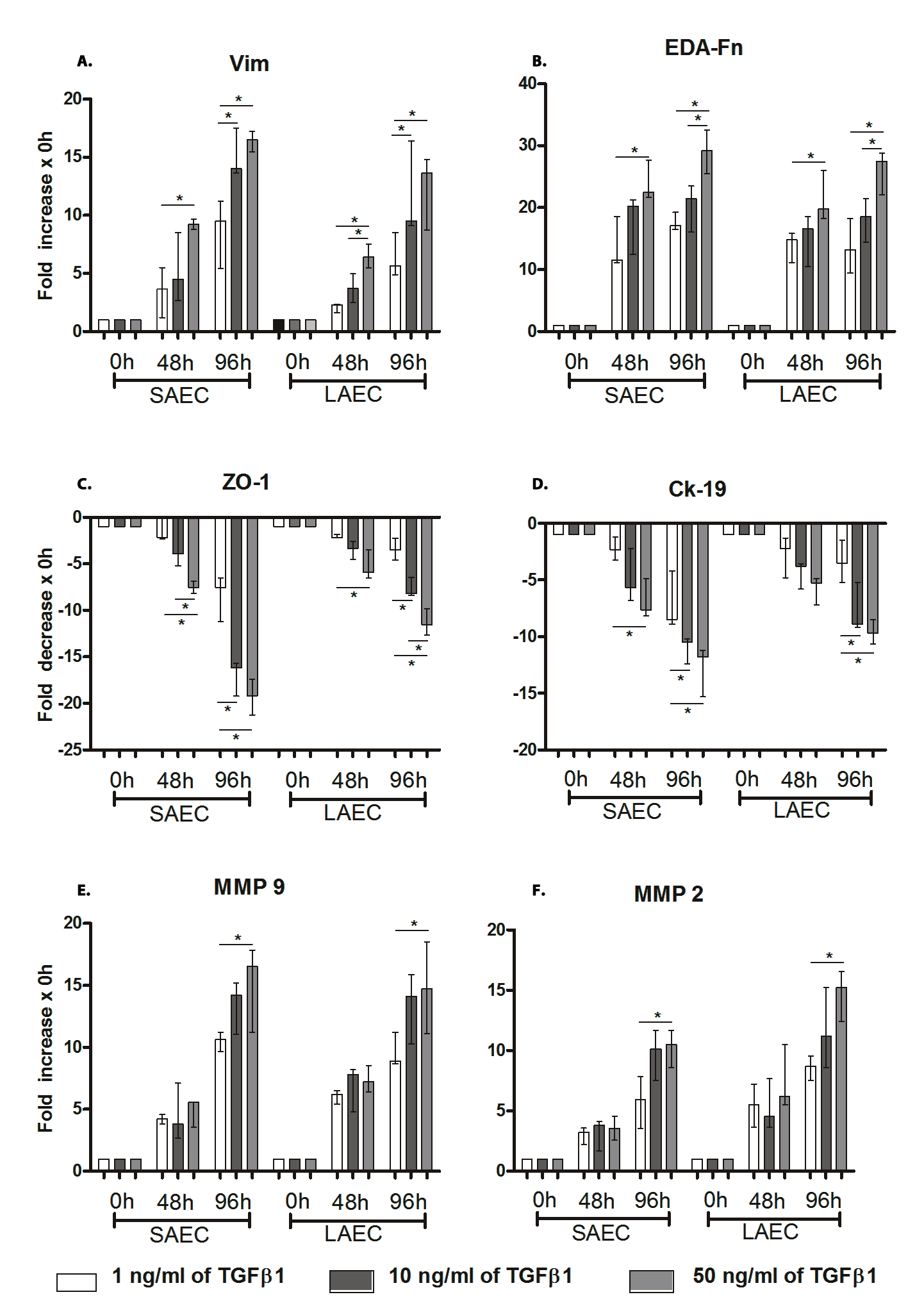

Supplement: Figure S1 — The ability of TGFβ1 to induce EMT in the primary human allograft SAEC and LAEC was compared at 3 different concentrations of TGFβ1–1 ng/ml, 10 ng/ml and 50 ng/ml (n = 4). The change in the expression of mesenchymal markers (A) Vimentin (Vim), (B) EDA- Fibronectin (EDA-Fn) and epithelial marker (C) Zona Occludens -1 (ZO-1), (D) Cytokeratin-19 (Ck-19) was measured by Western blots and change in (E) MMP -2 and (F) MMP -9 activity was measured by gelatin zymography. At 96 h post stimulation there was a decrease in the expression of epithelial markers, and increase in the expression of mesenchymal markers as well as MMP-2 and −9, as typically observed during EMT for all doses studied. A clear dose-response relationship was observed, with the most pronounced change in protein expression observed when cultures were stimulated with 50 ng/ml of TGFβ1. No cell loss was observed for any of the cultures. For future studies 50 ng/ml of TGFβ1 was chosen as the optimal concentration to induce EMT after 96 h of in vitro stimulation in SAEC and LAEC. Data presented as median ± IQR. *p<0.05 (TIF) [file pone.0052309.s001.tif]

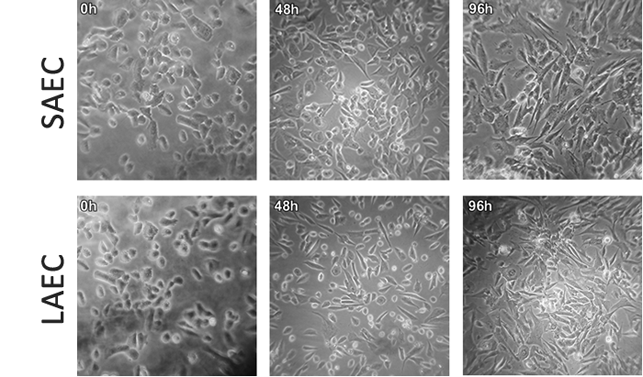

Supplement: Figure S2 — Morphological changes in small airway epithelial cells (SAEC) and large airway epithelial cells (LAEC) when stimulated with TGFβ1 (50 ng/ml) for 96 h in vitro. SAEC and LAEC cell cultures were stimulated with TGFβ1 and were photographed using phase contrast microscopy at 0 h, 48 h and 96 h. Unstimulated (left column) SAEC (top row) and LAEC (bottom row) exhibit typical polygonal, cobblestone morphology. Upon stimulation with TGFβ1, cells were observed to elongate and become spindle-shaped. After 96 h, cells assumed the typical mesenchymal phenotype, consistent with epithelial-mesenchymal transition (EMT). (400x magnification). (TIF) [file pone.0052309.s002.tif]

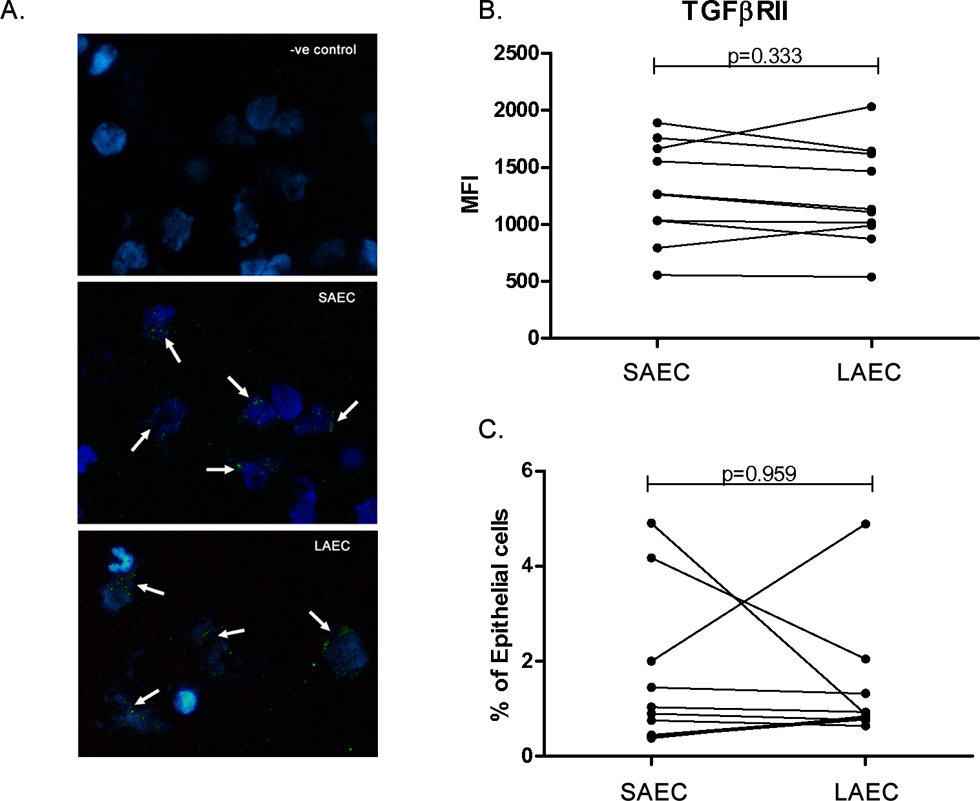

Supplement: Figure S3 — To test whether the increased susceptibility of SAEC to EMT was due to an increased expression of TGFβRII, ex vivo samples from small and large airway brushings were analysed for protein expression of TGFβRII using immunocytochemistry (A, n = 6, 400x magnification) and flow cytometry (B&C, n = 8). Figure S3B demonstrates the mean fluorescent intensity (MFI) for TGFβRII, and Figure S3C demonstrates the proportion of epithelial cells expressing TGFβRII. For immunocytochemistry epithelial cells were cytospun onto glass slides and stained for TGFβRII expression (green, indicated by arrows) as well as the nuclear stain DAPI (blue). For flow cytometry, cells were stained with, TGFβRII-PE and counterstained with cytokeratin-FITC to confirm epithelial lineage. There was no difference in either the intensity of expression or the proportion of epithelial cells expressing the TGFβRII. (TIF) [file pone.0052309.s003.tif]

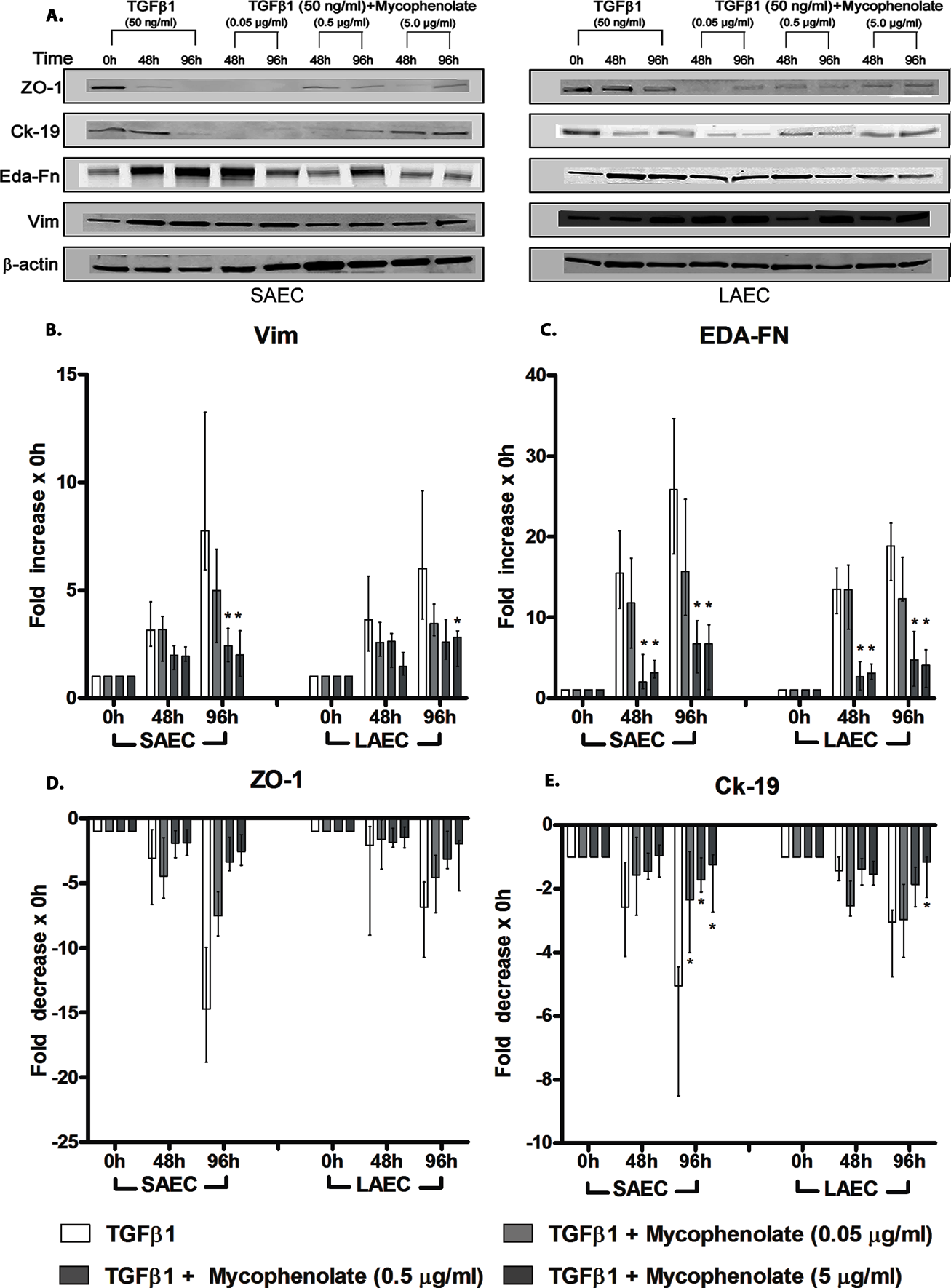

Supplement: Figure S4 — Effect of Mycophenolate on TGFβ1-induced EMT of primary human small and large airway epithelial cell cultures – expression of epithelial and mesenchymal markers. Primary small airway epithelial cell (SAEC) and large airway epithelial cell (LAEC) cultures were stimulated with TGFβ1 (50 ng/ml) for 96 h to induce EMT. Mycophenolate (5 µg/ml) was simultaneously added to observe its effect of preventing or slowing EMT. (A) Initial immunoblots suggested that addition of mycophenolate suppressed EMT in a dose dependant manner. Further quantification of immunoblots showed a marked increase in mesenchymal markers (B) Vimentin, (C) EDA-Fibronectin and corresponding decrease in epithelial markers (D) Zona Occludens -1, (E) Cytokeratin-19 after 96 h of stimulation. Addition of mycophenolate at the highest dose (5 µg/ml) significantly suppressed the increase in expression of mesenchymal markers and the decrease in expression of epithelial markers in SAEC and LAEC, observed in the control samples which were stimulated with TGFβ1 alone (n = 6). Data presented as median ± IQR. *p<0.05 compared to control. (TIF) [file pone.0052309.s004.tif]

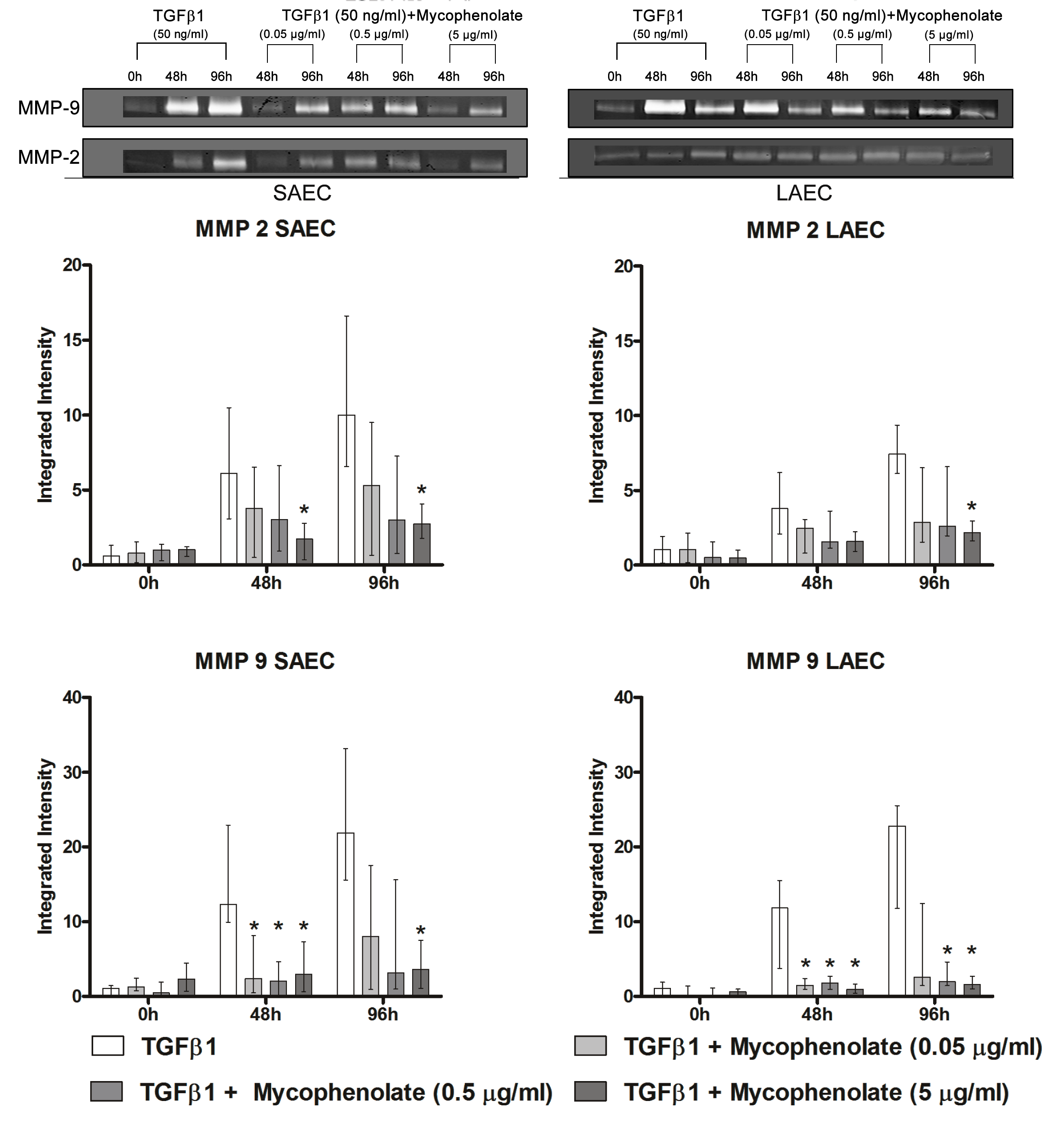

Supplement: Figure S5 — Effect of Mycophenolate on TGFβ1-induced EMT of primary human small and large airway epithelial cell cultures - activity of matrix metalloproteinases (MMP) -2 & -9. Primary small airway epithelial cell (SAEC) and large airway epithelial airway cell (LAEC) cultures were stimulated with TGFβ1 (50 ng/ml) for 96 h to induce EMT. Mycophenolate (5 µg/ml) was simultaneously added to observe its effect on preventing or slowing EMT. (A) Initial zymograms suggested a suppression of EMT in a dose dependant manner. The suppression of EMT associated increase in gelatinolytic activity of (B&C) MMP-2 and (D&E) MMP-9 by mycophenolate was further quantified. Addition of mycophenolate at the highest dose (5 µg/ml) suppressed the increase in the activity of matrix metalloproteinase (MMP) 2 and 9 in SAEC and LAEC, observed in the control samples which were stimulated with TGFβ1 alone (n = 6). Data presented as median ± IQR. *p<0.05 compared to control. (TIF) [file pone.0052309.s005.tif]

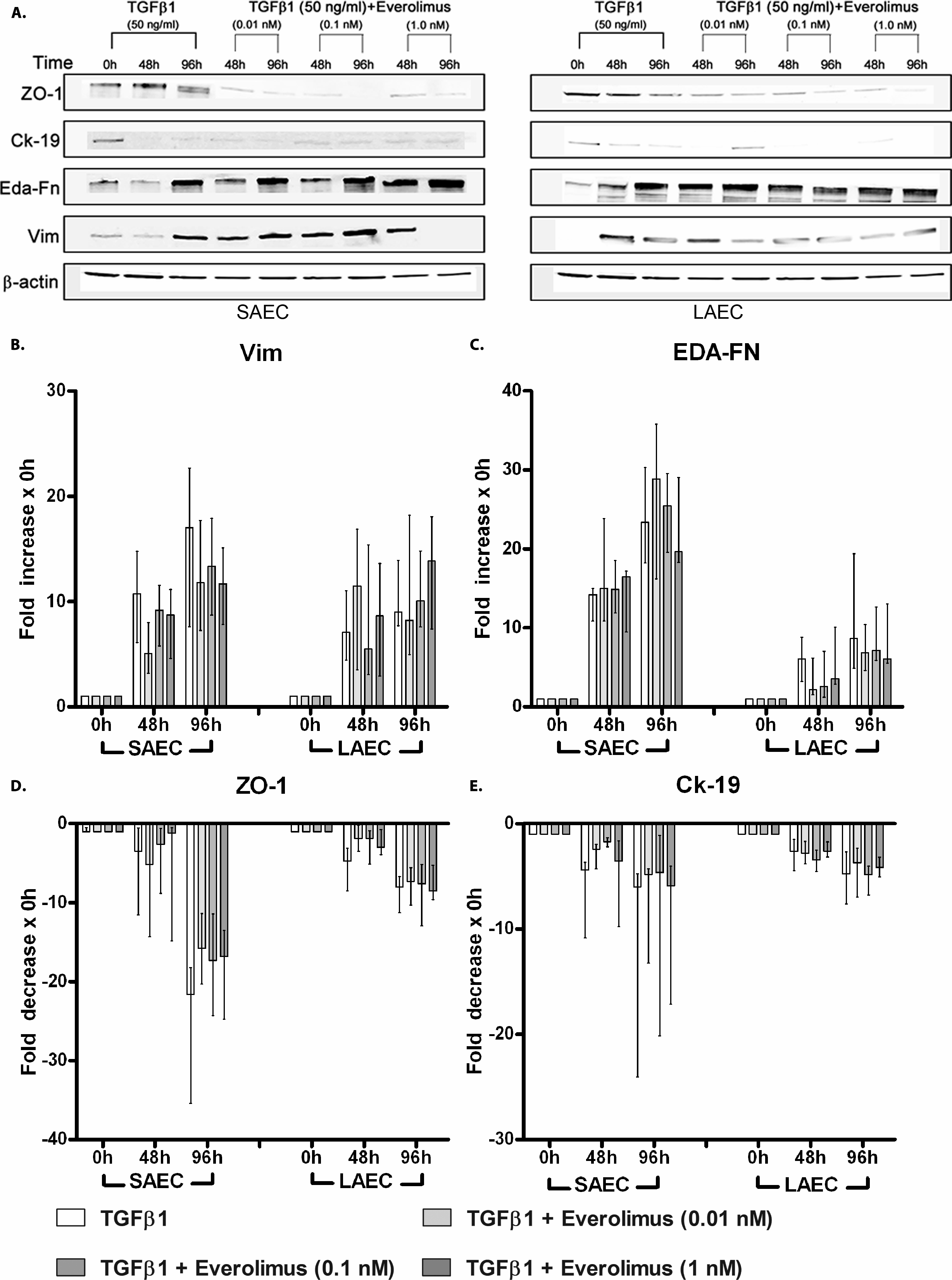

Supplement: Figure S6 — Everolimus was unable to prevent TGFβ1 (50 ng/ml)-induced of small airway epithelial cell (SAEC) and large airway epithelial cell (LAEC) cultures. Primary small (SAEC) and large (LAEC) airway epithelial cell cultures were stimulated with TGFβ1 (50 ng/ml) for 96 h to induce EMT. Everolimus was simultaneously added to observe its effect of preventing or slowing EMT. (A) Initial immunoblots suggested that addition of everolimus at all three doses (0.01 nM, 0.1 nM, 1 nM) did not significantly suppress EMT. Further quantification of immunoblots showed differences in expression of mesenchymal markers (B) Vimentin, (C) EDA-Fibronectin and epithelial markers (D) Zona Occludens -1, (E) Cytokeratin-19 after 96 h of stimulation, compared to control samples which were stimulated with TGFβ1 alone. (n = 6) Data presented as median ± IQR. *p<0.05 compared to control. (TIF) [file pone.0052309.s006.tif]

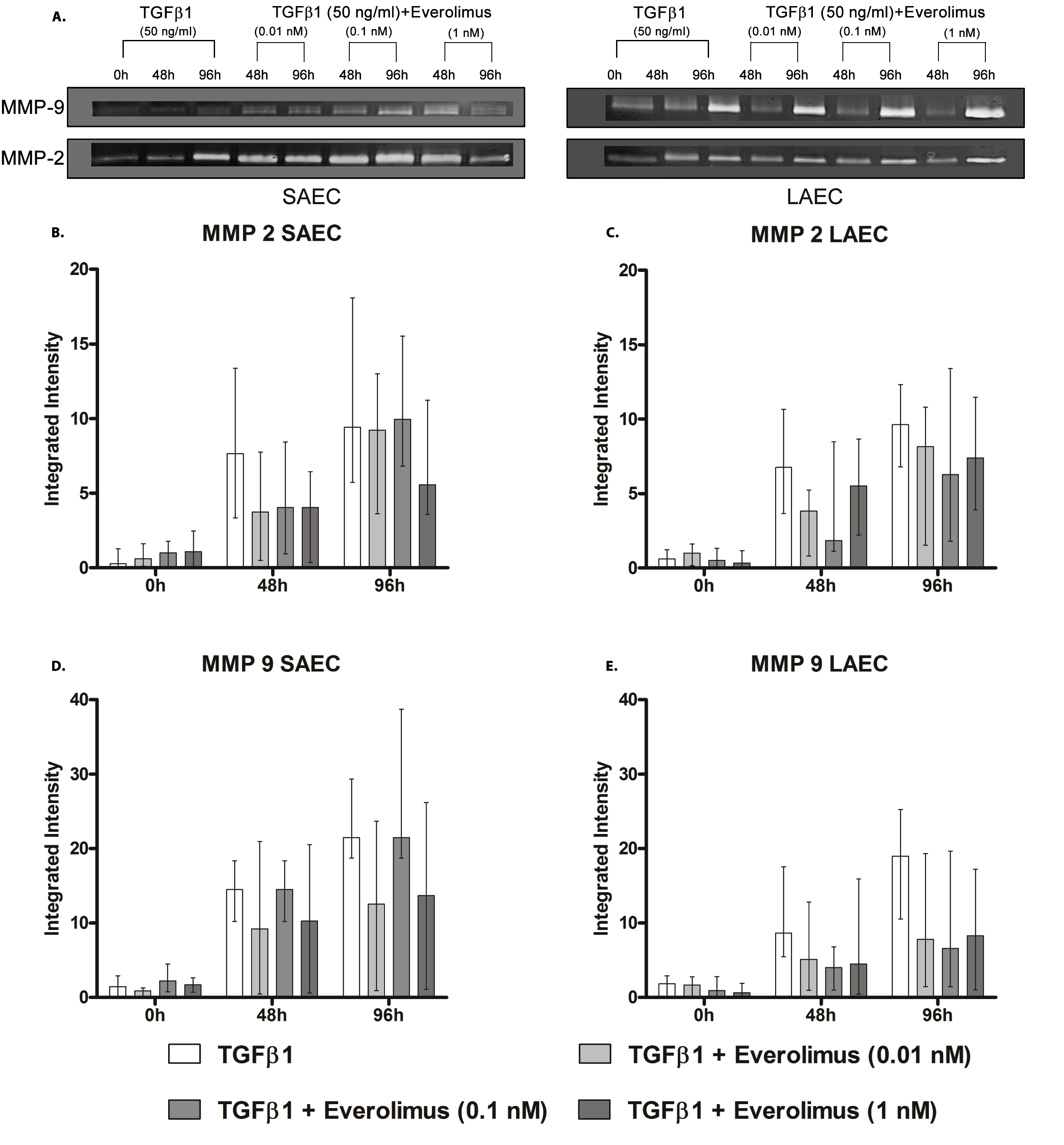

Supplement: Figure S7 — Addition of everolimus had no significant effect on the increase in the activity of matrix metalloproteinases (MMP) 2 & 9 associated with EMT. Primary small airway epithelial cell (SAEC) and large airway epithelial cell (LAEC) cultures were stimulated with TGFβ1 (50 ng/ml) for 96 h to induce EMT. Everolimus was simultaneously added to observe its effect on preventing or slowing EMT. (A) Initial zymograms suggested no suppression of EMT. The change in gelatinolytic activity of (B&C) MMP-2 and (D&E) MMP-9 by mycophenolate was further quantified. Addition of everolimus at all three doses (0.01 nM, 0.1 nM, 1 nM) did not significantly change the increase in the activity of matrix metalloproteinase (MMP) 2 and 9 in SAEC and LAEC, compared to control samples which were stimulated with TGFβ1 alone (n = 6). Data presented as median ± IQR. *p<0.05 compared to control. (TIF) [file pone.0052309.s007.tif]

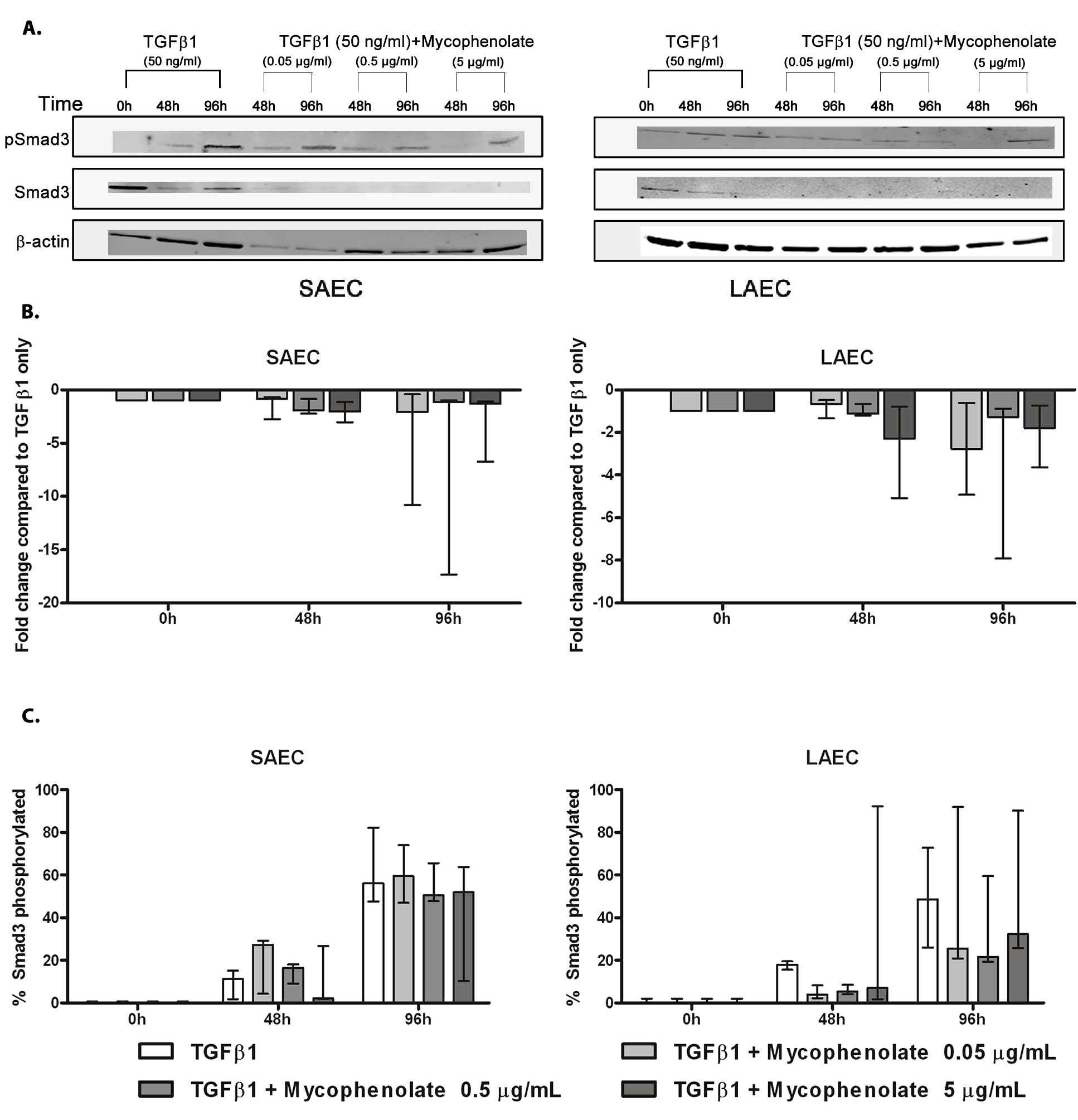

Supplement: Figure S8 — Mycophenolate partially prevented EMT by suppressing the expression of Smad3, but not Smad3 phosphorylation. Primary small (SAEC) and large (LAEC) airway epithelial cell cultures were stimulated with TGFβ1 (50 ng/ml) for 96 h to induce EMT. Mycophenolate was simultaneously added to observe its effect of preventing or slowing EMT. (A) Protein expression of Smad3 and pSmad3 was measured by Western blots. (B) Addition of mycophenolate (0.05 µg/ml, 0.5 µg/ml, 5 µg/ml) partially suppressed the expression of total Smad3 (Smad3+ pSmad3) in SAEC and LAEC, relative to the control cultures stimulated with TGFβ1 alone. This effect was particularly noticeable at the highest dose (5 µg/ml) (C) However addition of mycophenolate at all doses (1 µg/ml, 10 µg/ml, 50 µg/ml) did not alter the rate of Smad3 phsophorylation compared to TGFβ1 alone (n = 3) Data presented as median ± IQR. (TIF) [file pone.0052309.s008.tif]
